# 

**Published:** 1935

**Authors:** 


					Vol. LU. No. 195.0
.
q - .
<*?*. 4.
' H
SPRING, 1935.
k , v
# ? - . ?? -< y''V'-
V. /
mm
, m ??
The Bristol
Medico-Chirurgical Journal
'
PUBLISHED UNDER THE AUSPICES OP
THE BRISTOL MEDICO-CHIRURGICAL SOCIETY.
Editor : J. A. NIXON, C.M.G., F.R.C.P.
WITH WHOM ABE ASSOCIATED
? ? ? ' 1 V- ? 1
E. W. HEY GROVES, D.So., M.S., P.R.CJ.S. "
A. E. ILES, O.B.E., M.B., B.S., F.R.O.S.
A. RENDLE SHORT, M.D., B.S., B.Sc., P.R.C.S.
E. WATSON-WILLIAMS, M.C., Ch.M., P.R.O.S., Assistant] Editof.' / ?
Editorial Secretary: A. L. FLEMMING, M.B.; Cb.B.
3 ^mmhkbbSwr Srafcsa&fflBlaSggffiS&ra Jjrafilv ^SwaRsBe
* ~ *.'? ? ? ... ? M , > ? ' i 4
Hsmm.
BRISTOL: J. W. ARROWSMITH LTD., U QUAY STREET.
London : J. W. Asbowsmith (London) Ltd., 8 Endblbuqh Gabdkns, W.C. 1.
Price Three Shillings net.

				

